# Medical History from the Earliest Times

**Published:** 1892-11-12

**Authors:** E. T. Withington


### MEDICAL HISTORY FROM THE EARLIEST TIMES.

By E. T. Withington, M.A., M.P. Oxon.

### XXXI.—ARABIC MEDICINE. (4) THE WESTERN CALIPHATE.

Under the prosperous rule of Abdur-rahman III., al-Hakem II., and al-Mansur the Regent (912-1002), Cordova, capital of the Western Caliphate, was worthy of its ancient Phœnician name, Carta-Tuba, the great city. Its 200,000 houses may have contained a million inhabitants, whose wants, bodily, spiritual, and mental, were not unfairly provided for by 600 inns, 900 baths, 600 mosques, each with its free school attached, and including the whole province, 17 universities, and 70 public libraries. The library of al-Hakem might have vied with that of Alexandria, for, according to the lowest estimate, it contained 225,000 volumes, each of which had been read and annotated by the Caliph himself, if we may believe an admiring chronicler, who seems to forget that this implies a voracity of six volumes daily for a century. In an age when nobles and ladies were unable to sign their own names, there was hardly a boy or girl over twelve in the whole city and province who could not both read and write, and the adventurous Christian who penetrated to Cordova in pursuit of "Saracenic studies," rarely escaped being accused of unlawful dealings with those powers of darkness by whose aid alone the infidels could have acquired such wisdom. Michael the Scot is well known to readers of the modern Wizard of the North. Gerbert the Frank studied medicine among other things in Spain, and taught it at Rheims. We shall meet him again as Pope Sylvester II., and shall find that not even the chair of St. Peter could save one of its most learned and pious occupants from being considered, in a peculiar and terrible manner, the servant of Satan.

Such a civilisation could not fail to produce physicians and philosophers comparable to those of the East, and Rhazes, Haly Abbas, and Avicenna might have found worthy compeers in Albucasis, Avenzoar, and Averröes. The first of these, Abulkasem Khalaf ben Abbas, of Zahra, near Cordova, is sometimes placed last in order of time, but there is good reason to believe that he is the Khalaf ben Abbas who was physician to al-Hakem II., and that Leo Africanus is, for once, correct in saying that he died A.D. 1013, at the age of 101. He wrote a great medical cyclopædia in thirty books, called the "Tasrif," or "Method," but this would only deserve mention as one of the numerous similar works superseded by the Canon of Avicenna were it not for the last, or surgical book, which was published separately, and which marks an epoch in medical history. It was at once the first independent work on surgery, and the first illustrated treatise on that art. The practitioners of the thirteenth century borrowed from it far more frequently than they cared to confess, and Guy of Chauliac, the greatest of mediæval surgeons, quotes it more than two hundred times. Albucasis begins by saying that Arabic surgery is in a bad way owing to the ignorance of anatomy. "I have seen a surgeon incise a scrofulous swelling in a woman's neck; he stuck his knife into the cervical artery, and the patient fell dead in his arms. I have seen another extract a large stone; he got out the stone, but brought part of the bladder with it; the patient died on the third day." "Surgical operations," he continues, "are of two kinds, those which benefit the patient, and those which usually kill him," and he intends that a description of the dangers to be feared in various procedures shall form a special feature of his work. The first of the three chapters is entirely occupied with the manifold uses of the cautery, the favourite instrument of the Arab surgeon, and the book ends as it began, with his favourite watchword, "Caution!" "Avoid perilous practices, as I have already warned you, so shall you have the more praise and profit, if God will. . . This is the end of the book called al-Tasrif, written for those who have not the entire works of Abulkasem Khalaf ben Abbas the Zahraite, on whom may God have mercy if he finishes his life in the good way."

Christian writers split up Abulkasem into at least three individuals, whom they called Albucasis, Alsaravius, and Benabasarem respectively, while, on the other hand, they joined several members of the great medical family of Ebn Zohr into one, whence arose the legend of a physician of that name who lived 137 years. The only one who can be here considered is Abu Merwan Abdalmalek ebn Zohr, the greatest physician of the twelfth century, and, next to Rhazes, the most original of the Arabs. His chief work, the "Theisir," or "Assistance," contains much which is not to be found in earlier writers, for, besides describing pericarditis and mediastinal abscess, Avenzoar may perhaps claim the distinction of discovering the Acarus scabiei, or itch mite. In the nineteenth chapter of the second book he says: "Sometimes there arise on the body, under the external skin, little swellings which the vulgar call 'soab,' and if the skin be removed there issues from various parts a very small beast, so small that he is hardly visible." But, "through the goodness of God," there are many things which will cure this disease, and though Avenzoar does not mention sulphur, he specially recommends an application containing oil of bitter almonds. Avenzoar, like his father, was in high favour with the Emir of Seville, who, during the persecution of the heterodox, gave him a commission outside the city. He was accused in his absence of possessing improper books. "By Allah," cried the Emir, "I sent him away that he might escape the attacks of his enemies. Though this charge were signed by all my subjects, I would not believe it; I know what a religious man he is."

The "Theisir" of Avenzoar is usually bound up with the "Colliget" of his friend and pupil Averröes. The Arabic title of this work, "Kotab ol Kollyat," or Book of Universals, sufficiently indicates its philosophic character, and Averröes declared he desired no higher title than that of commentator on the writings of Aristotle. He had, however, an important influence both on the physic and philosophy of the middle ages, and we may again refer to him when describing the scholastic medicine of the thirteenth century. Averröes is the last of the great Arab physicians, and the decline of science was due to two closely connected causes, political decadence and the revival of orthodoxy. It was because they had forgotten God, said the Moslem theologians, that the warriors of Islam turned their backs in the day of battle; and they remembered Averröes. That free-thinking philosopher was degraded from his high office of Viceroy of Cordova and banished to a town inhabited only by Jews, while, if we may trust the doubtful evidence of Leo Africanus, he was also compelled to stand at the door of a mosque, where every passer-by spat in his face. A still more legendary story relates that when told that both Christians and Moslem had devoted his books to temporal and his soul to eternal flames, he made the famous answer, "Sit anima mea cum philosophis," or its Arabic equivalent.

The orthodox reaction affected another physician, almost as famous as Averröes. Moses ben Maimon (1135—1204), now known as the Rabbi Maimonides, was studying medicine at Cordova, when the decree went forth that all Jews and Christians must accept Islam or leave the country. He conformed outwardly until he had arranged his affairs, and then went to Egypt, where he became a member of the medical staff of the Sultan Saladin, which comprised Christians, Jews, and Moslem indiscriminately. He was soon accused of apostasy from the faith, a crime which the Koran punishes with death, but Saladin's judges, with a liberality worthy of their master, acquitted him, declaring that a forced conversion is no conversion.

The medical works of Maimonides, which alone concern us here, are written in the tedious semi-philosophic style of Avicenna and Averröes, with the exception of the "Book of Counsel" addressed to the Sultan Malek al Afdhal ben Saladin, which is of great interest, and forms the earliest example of a form of medical literature of which we shall have much to say. Here is the preface: "There having come to the hands of the least of his slaves, Moses the Israelite of Cordova, a command from the high and mighty Prince Malek, whom may God increase in honour, that I should write instructions as to regimen by which he may safely and surely be freed from the cares and sickness that have come upon him, I pray that God will deign to remove these and all other disorders, and that perfect health may attend him all the days of his life. It has been signified to me that my revered Lord complains of the hardness and dryness of his nature, whereby he cannot get natural relief without much straining and difficulty; that he is oppressed by melancholy thoughts; that he seeks to be alone, and is in fear of death; and that the weakness of his stomach prevents the proper digestion of food. Wherefore I have written the four following tractates. The first is a brief explanation of the general rules of health; the second is for those sick persons who cannot find a physician, or at least not one whom they can trust; the third contains the regimen proper for my Lord's case, as it has been described to me; the fourth treats of matters useful to sick and well in all times and places. And let none blame me if I repeat here what I have said elsewhere, for each work must be adapted to the needs of those who require it, and to the objects of the writer. And for these things I now implore the aid and inspiration of God, the glorious, the great."

